# Optogenetic Manipulation of Cerebellar Purkinje Cell Activity *In Vivo*

**DOI:** 10.1371/journal.pone.0022400

**Published:** 2011-08-05

**Authors:** Tadashi Tsubota, Yohei Ohashi, Keita Tamura, Ayana Sato, Yasushi Miyashita

**Affiliations:** 1 Department of Physiology, The University of Tokyo School of Medicine, Bunkyo-ku, Tokyo, Japan; 2 Department of Physics, The University of Tokyo School of Science, Bunkyo-ku, Tokyo, Japan; Chiba University Center for Forensic Mental Health, Japan

## Abstract

Purkinje cells (PCs) are the sole output neurons of the cerebellar cortex. Although their anatomical connections and physiological response properties have been extensively studied, the causal role of their activity in behavioral, cognitive and autonomic functions is still unclear because PC activity cannot be selectively controlled. Here we developed a novel technique using optogenetics for selective and rapidly reversible manipulation of PC activity *in vivo*. We injected into rat cerebellar cortex lentiviruses expressing either the light-activated cationic channel channelrhodopsin-2 (ChR2) or light-driven chloride pump halorhodopsin (eNpHR) under the control of the PC-specific L7 promoter. Transgene expression was observed in most PCs (ChR2, 92.6%; eNpHR, 95.3%), as determined by immunohistochemical analysis. *In vivo* electrophysiological recordings showed that all light-responsive PCs in ChR2-transduced rats increased frequency of simple spike in response to blue laser illumination. Similarly, most light-responsive PCs (93.8%) in eNpHR-transduced rats decreased frequency of simple spike in response to orange laser illumination. We then applied these techniques to characterize the roles of rat cerebellar uvula, one of the cardiovascular regulatory regions in the cerebellum, in resting blood pressure (BP) regulation in anesthetized rats. ChR2-mediated photostimulation and eNpHR-mediated photoinhibition of the uvula had opposite effects on resting BP, inducing depressor and pressor responses, respectively. In contrast, manipulation of PC activity within the neighboring lobule VIII had no effect on BP. Blue and orange laser illumination onto PBS-injected lobule IX didn't affect BP, indicating the observed effects on BP were actually due to PC activation and inhibition. These results clearly demonstrate that the optogenetic method we developed here will provide a powerful way to elucidate a causal relationship between local PC activity and functions of the cerebellum.

## Introduction

The functions of the cerebellum range from the control and coordination of movements to autonomic regulation [1]. Although the basic neuronal circuitry of the cerebellar cortex is uniform everywhere, there are functional differences between various parts of the cerebellar cortex, primarily owing to differences in input and output connectivity [2]. In past studies, lesioning, electrical stimulation, and chemical activation/deactivation have been used to investigate local functions of the cerebellar cortex [3]–[6]. However, these manipulations affect not only Purkinje cells (PCs), the sole output neurons of the cerebellar cortex, but also local excitatory, inhibitory, and modulatory cells, as well as non-local cells that send their axons to the cerebellar cortex. It is thus still unclear whether the effects of stimulation or lesions are actually due to the activation or absence (deactivation) of PCs. To elucidate the causal relationship between PC activity and a function of the cerebellum, therefore, a method for selective and rapidly reversible manipulation of PC activity is necessary.

Recently, optogenetic technologies have been developed and applied to various neuronal systems to manipulate the activity of selected neuronal populations in living animals [7]–[9]. Several light-responsive proteins have been reported, such as channelrhodopsin-2 (ChR2) and halorhodopsin (NpHR) [10], [11]. They have fast temporal kinetics that makes it possible to drive or inhibit the generation of action potentials at the millisecond timescale *in vivo* [8], [10], and they can target specific neuronal populations with cell-type specific promoters [12], [13]. Because there is a well-characterized PC-specific promoter, L7 [14], optogenetic proteins are an ideal tool for manipulating cerebellar cortical outputs.

Here, we developed a HIV1-derived lentiviral vector system to express ChR2 or enhanced NpHR (eNpHR) [15] specifically in cerebellar PCs under the control of a 1 kb L7 promoter. Immunohistochemical analysis showed that optogenetic proteins were expressed in PCs with high cell-type specificity. *In vivo* single-unit recordings of PCs revealed that almost all of the PCs that express ChR2 or eNpHR were activated or inhibited during blue or orange light illumination, respectively. Furthermore, we applied this system to the analysis of the cerebellar cardiovascular region situated within lobule IXb [16] to show how the manipulation of PC activity in opposite directions (activation or inhibition) affects BP in urethane-anesthetized rats. Although there is considerable evidence that electrical or chemical stimulation of lobule IXb evokes cardiovascular responses in cats and rabbits [5], [16]–[18], it is still not possible to determine the relationship between activity of PCs in lobule IXb and cardiovascular responses, because electrical or chemical stimulation cannot control PCs without affecting other neuronal types. We thus characterized this relationship using optogenetic PC activity manipulation, and showed that the ChR2-mediated photostimulation and eNpHR-mediated photoinhibition of lobule IXab in rats evoked depressor and pressor responses, respectively.

## Results

### Genetic targeting of ChR2- or eNpHR-expression to PCs

**Figure 1:**
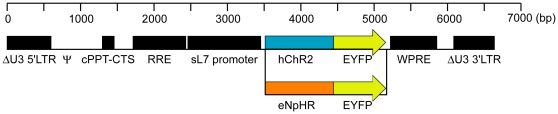
Schematic diagrams of the HIV1-derived SIN lentiviral vector constructs, Lenti-sL7-hChR2-EYFP-WPRE (sL7-ChR2) and Lenti-sL7-eNpHR-EYFP-WPRE (sL7-eNpHR). The 5′ LTR is chimeric in that the U3 region of the HIV-1 LTR is replaced with CMV enhancer. A deletion in the U3 region of the 3′ LTR renders it self-inactivating; in addition, a portion of the R region and the U5 region have been removed and replaced with the rabbit beta-globin gene polyadenylation site. The sL7 promoter (1 kb) was used to express the ChR2-EYFP or eNpHR-EYFP. LTR, long terminal repeat; ψ, packaging region; cPPT, central polypurine tract; CTS, central termination sequence; RRE, Rev-responsive element, WPRE, woodchuck hepatitis post-transcriptional regulatory element.

**Figure 2:**
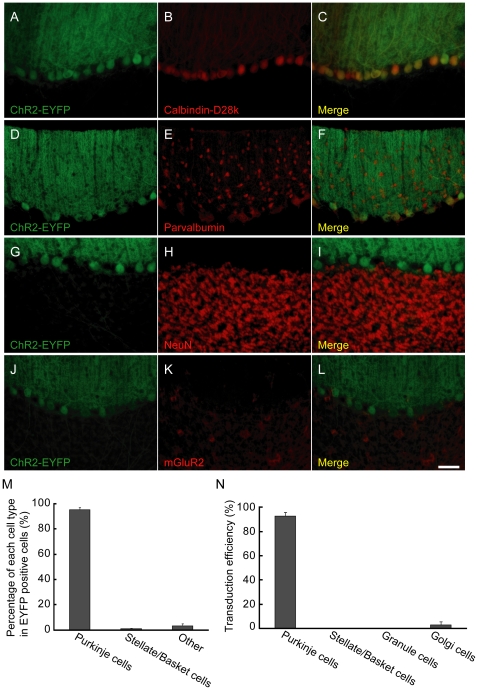
Immunostaining of a parasagittal section of sL7-ChR2 rat cerebellum. Four parasagittal sections (A–C, D–F, G–I, J–L) are shown. Sections with EYFP fluorescence (A, D, G, J) were counterstained for calbindin-D28k (B), parvalbumin (E), NeuN (H) and mGluR2 (K), respectively. The rightmost panels (C, F, I, L) show the merged image. In panel F, ChR2-EYFP negative/parvalbumin positive basket cell axons can be observed around each PC. Scale bar, 50 µm. (M) The percentages of PCs, stellate/basket cells and other cell types among all ChR2-EYFP positive cells. (N) Transduction efficiencies into each cell type. n = 4 rats.

We used the L7 (Pcp2) promoter [14], [19] to target optogenetic transgenes to PCs. The L7 promoter was shortened to 1 kb length, to load it into lentiviral vectors (abbreviated as sL7 promoter) [20]. Two lentiviral vectors were constructed, one of which was Lenti-sL7-hChR2-EYFP-WPRE, and the other one of which was Lenti-sL7-eNpHR-EYFP-WPRE (abbreviated as sL7-ChR2, sL7-eNpHR, respectively, Fig. 1). To investigate the expression pattern of ChR2-EYFP, cerebellar sections of sL7-ChR2-injected rats (sL7-ChR2 rats) were stained for calbindin-D28k, parvalbumin, NeuN and mGluR2, which are markers of Purkinje, stellate/basket, granule and Golgi cells [21], [22], respectively. Strong ChR2-EYFP expression was observed in PCs (Fig. 2A–C), but not in stellate/basket cells (Fig. 2D–F), granule cells (Fig. 2G–I) or Golgi cells (Fig. 2J–L). To compare ChR2-EYFP expression among different cell types, we calculated the percentage of each cell type among all ChR2-EYFP-positive cells. Almost all of the ChR2-EYFP-positive cells were PCs (95.0±2.1%, n = 4 rats, 12 sections, Fig. 2M). The percentages of stellate/basket cells and other cell types (potentially including granule, Golgi, Lugaro and unipolar brush cells) were quite low (0.9±0.5% for stellate/basket cells, 3.2±2.4% for other cell types, Fig. 2M). We also estimated the transduction efficiency into each cell type to examine how effectively ChR2-EYFP was expressed in each cell type. ChR2-EYFP expression was detected in most PCs (92.6±3.1%), but in only very small percentages of other cell types (0.2±0.0% for stellate/basket cells, 0.0% for granule cells, 2.8±2.8% for Golgi cells, Fig. 2N). These results show that the ChR2-EYFP expression induced by the sL7 promoter was highly specific to PCs.

**Figure 3:**
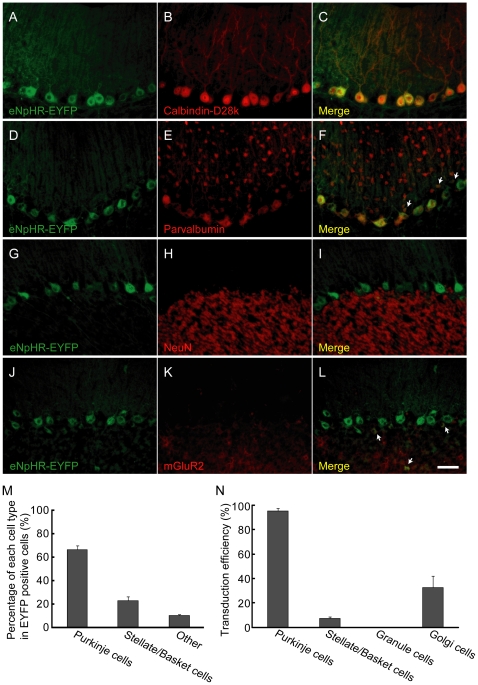
Immunostaining of a parasagittal section of sL7-eNpHR rat cerebellum. Four parasagittal sections (A–C, D–F, G–I, J–L) are shown. Sections with EYFP fluorescence (A, D, G, J) were counterstained for calbindin-D28k (B), parvalbumin (E), NeuN (H) and mGluR2 (K), respectively. The rightmost panels (C, F, I, L) show the merged image. Arrows in panel F and L indicate eNpHR-EYFP positive/parvalbumin positive basket cells and eNpHR-EYFP positive/mGluR2 positive Golgi cells, respectively. Scale bar, 50 µm. (M) The percentages of PCs, stellate/basket cells and other cell types among all eNpHR-EYFP positive cells. n = 8 rats. (N) Transduction efficiencies into each cell type. n = 8 rats for Purkinje, stellate/basket and granule cells. n = 7 rats for Golgi cells.

We next examined the expression pattern of eNpHR-EYFP in sL7-eNpHR-injected rats (sL7-eNpHR rats). As in the case of ChR2, strong eNpHR-EYFP expression was observed in PCs (Fig. 3A–C). However, there was also moderate expression of eNpHR-EYFP in both stellate/basket cells and Golgi cells (indicated by arrows, Fig. 3D–F, J–L). No eNpHR-EYFP-positive granule cells were observed (Fig. 3G–I). The percentages of PCs, stellate/basket cells and other cell types among eNpHR-EYFP-positive cells were 66.5±3.5, 23.1±3.2 and 10.3±0.7%, respectively (n = 8 rats, 24 sections for Purkinje, stellate/basket and granule cells. n = 7 rats, 21 sections for Golgi cells, Fig. 3M). Transduction efficiencies into PCs, stellate/basket cells, granule cells and Golgi cells were 95.3±1.9, 7.1±1.7, 0.0 and 32.7±9.4%, respectively (Fig. 3N). These results suggest that the eNpHR-EYFP expression induced by the sL7 promoter was less specific for PCs than the ChR2-EYFP expression induced by the same promoter, although the transduction efficiency into PCs was significantly higher than that into other cell types (*P*<0.01, Tukey-Kramer multiple comparison test).

### Electrophysiological characterization of ChR2- or eNpHR-expressing PC responses with light illumination *in vivo*

**Figure 4:**
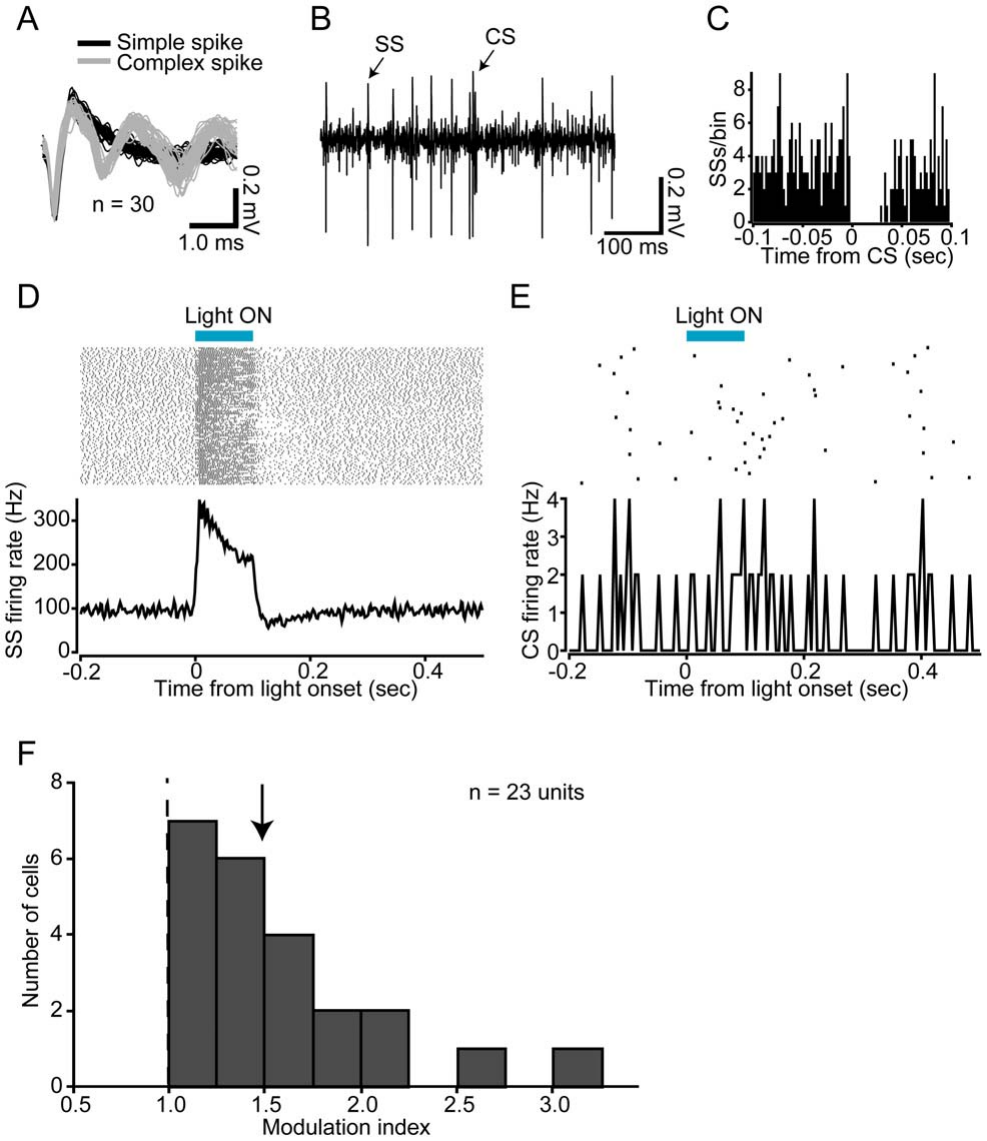
Electrophysiological recordings of PC activity in response to blue light illumination in sL7-ChR2 rats. (A) Waveforms of simple spikes (SSs, black lines) and complex spikes (CSs, gray lines). (B) A sample record of spontaneous activity is shown. Transient pause of SS firing was observed after a CS firing. (C) Peri-CS time histogram of SSs. This histogram indicates discharge rate of SSs before and after the occurrence of a CS. Bin width of the histogram is 1 ms. (D) Responses of SSs to blue light illumination. Shown at the top is a spike raster plot (100 trials are shown in horizontal row), and at the bottom is a peri-stimulus time histogram (PSTH), averaged across all trials (bin width, 2 ms). Period of blue light illumination is indicated by a horizontal blue bar (100 ms duration). (E) Same as D, but of CSs. Bin width of the PSTH is 5 ms. (F) A histogram of modulation index (MI) of SS firing rate. MI of each light-responsive cell was calculated as follows: (firing rate during the 50 ms period before light onset)/(firing rate during the 50 ms period after latency). An arrow indicates the median value. n = 23 units, 4 rats.

To test the functional expression of optogenetic transgenes in PCs, we performed electrophysiological single-unit recordings and examined how the firing rates of light-responsive PCs in sL7-ChR2 or sL7-eNpHR rats are affected by blue or orange light illumination *in vivo*. The blue light source used for illuminating sL7-ChR2 rat cerebellum was a DPSS laser (peak wavelength at 473 nm). We considered a neural signal to originate from a PC if it exhibited two types of spiking activities: simple spikes (SSs) and complex spikes (CSs) (Fig. 4A). SSs and CSs were judged to originate from the same PC when a transient pause (∼20 ms) in SS firing followed each CS in the raw trace (Fig. 4B), and the peri-CS histogram of SSs (Fig. 4C) [23], [24]. In this study, we recorded only single units identified as PCs. When a PC in sL7-ChR2 rat was illuminated by blue light, strong and time-locked elevation of SS firing rate was observed (Fig. 4D). In contrast, CS firing was not affected by blue light illumination (Fig. 4E). We recorded total of 23 light-responsive PCs (n = 4 rats), and a histogram of their SS firing rate modulation levels are shown in Fig. 4F. All increased SS firing rate in response to blue light (median of modulation index (MI) = 1.49), and some were strongly activated to above 100% compared to spontaneous firing rates.

**Figure 5:**
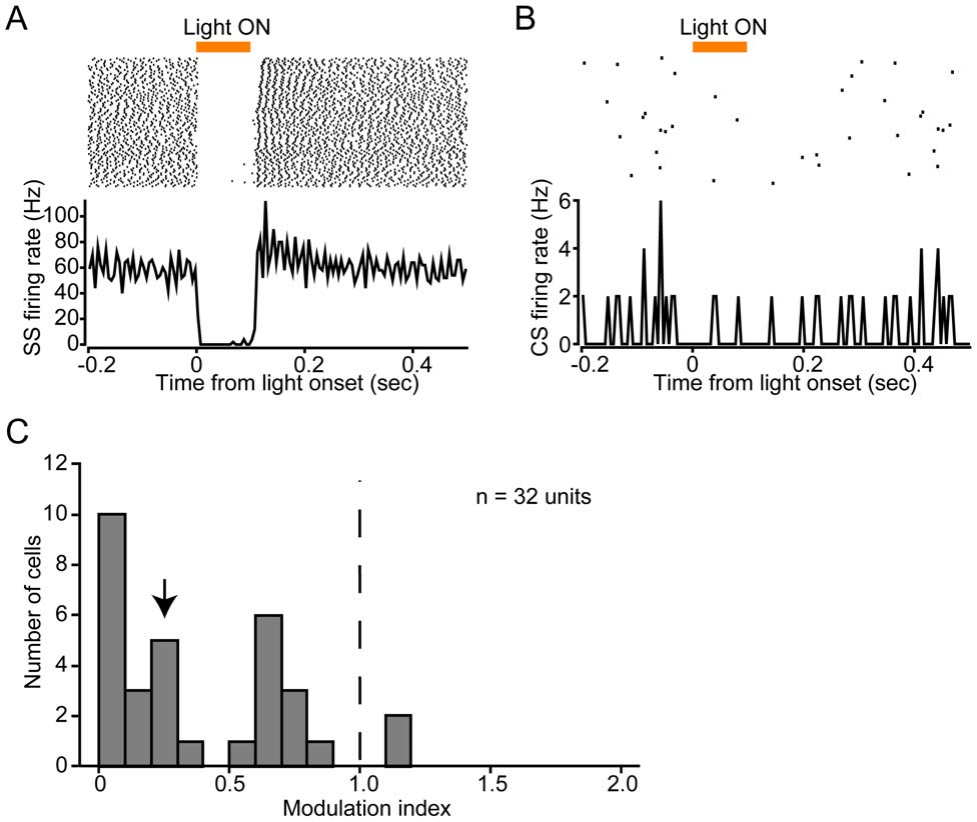
Electrophysiological recordings of PC activity in response to orange light illumination in sL7-eNpHR rats. (A) Responses of SSs. Shown at the top is a spike raster plot (100 trials are shown in horizontal row), and at the bottom is a PSTH, averaged across all trials (bin width, 2 ms for simple spikes). Period of orange light illumination is indicated by a horizontal orange bar (100 ms duration). (B) Same as A, but of CSs. Bin width of the PSTH is 5 ms. (C) A histogram of MI of SS firing rate. An arrow indicates the median value. n = 32 units, 7 rats.

For electrophysiological recordings of light-responsive PCs in sL7-eNpHR rats during orange light illumination, we used another DPSS laser (peak wavelength at 593 nm). The activity of a representative light-responsive PC is shown in Fig. 5A and B. This cell strongly decreased SS firing rate in response to orange light (Fig. 5A), though eNpHR-mediated inhibition did not affect CSs (Fig. 5B). We recorded from a total of 32 light-responsive PCs (n = 7 rats), and their SS firing rate modulation levels are shown in Fig. 5C. Most of them decreased SS firing rate in response to orange light (median MI = 0.25). Although two cells slightly increased SS firing rate, SS modulation levels of these cells were relatively weak, and the percentage among all light-responsive PCs was also small (6.3%, Fig. 5C). We therefore concluded that population PC activity in sL7-eNpHR rats is strongly inhibited by orange light illumination.

### ChR2-mediated photostimulation of PCs in the uvula provokes depressor responses

**Figure 6:**
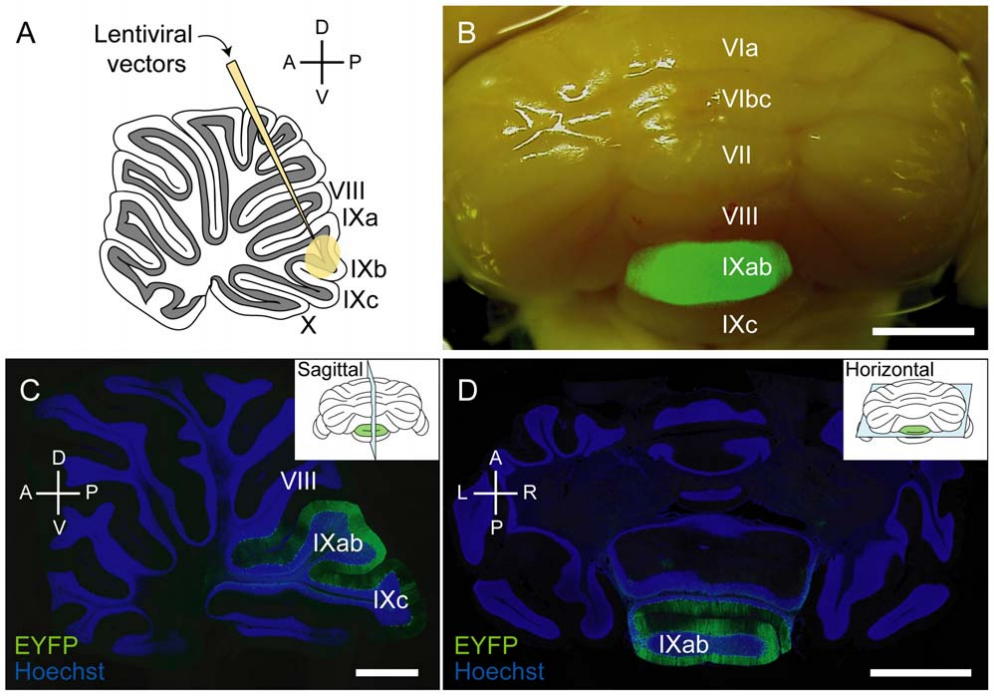
Lentiviral vector-mediated gene transfer into lobule IXab of rat cerebellum. (A) Schematic diagram of lentiviral vector injection into lobule IXab. (B) A representative stereoscopic fluorescent image of a cerebellum injected with sL7-eNpHR. Scale bar, 2 mm. (C, D) A representative sagittal section (C) and horizontal section (D) of lentiviral vector-injected rat cerebellum. The inset of each panel shows cutting plane of each section. EYFP fluorescence was detected mainly in the lobule IXab. Scale bars, 2 mm (B, D) and 1 mm (C).

**Figure 7:**
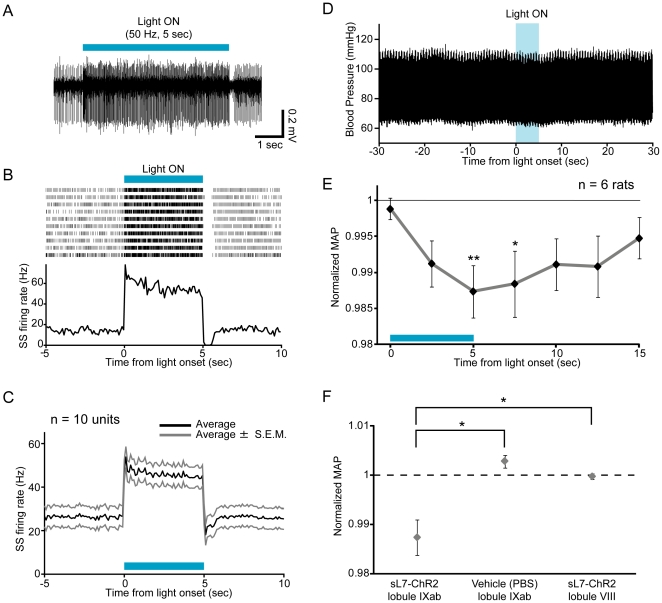
The effects of ChR2-mediated photostimulation of PCs in lobule IXab. (A) Photostimulation of lobule IXab by 5 sec light train (indicated by a horizontal blue bar) increased firing rate of a PC. Blue light was illuminated at 50 Hz (10 ms illumination/10 ms interval). (B) Responses of SSs to photostimulation. Shown at the top is a spike raster plot (10 trials are shown in horizontal row), and at the bottom is a PSTH, averaged across all trials (bin width, 100 ms). The intertrial interval was 90 sec. Period of photostimulation is indicated by a blue bar. (C) Averaged response of SSs. Black line indicates average, and gray lines indicate average ± SEM. n = 10 units, 2 rats. (D) A sample raw trace of arterial blood pressure around the time of photostimulation of lobule IXab. (E) BP response to photostimulation averaged across rats. *P<0.05, **P<0.01, one-way repeated measures ANOVA followed by Bonferroni-corrected *t*-test. Width of time window used for calculating nMAP values at each time point is 1 sec. n = 6 rats. (F) The effects of photostimulation of lobule IXab on BP were compared with that of blue light illumination onto vehicle (PBS)-injected lobule IXab (n = 3 rats) and photostimulation of lobule VIII (n = 4 rats). nMAP values averaged during the period from 4 to 5 sec after light illumination onset were plotted. *P<0.05, Tukey-Kramer multiple comparison test.

We next examined whether ChR2-mediated photostimulation of PCs within the cerebellar uvula evokes depressor responses as well as electrical stimulation in anesthetized animals [16]. The lentiviral vectors were targeted to lobule IXab (Fig. 6A). Transgene expression in lobule IXab was confirmed by stereoscopic fluorescence microscope analysis (Fig. 6B), and also by immunohistochemical analysis of sagittal (Fig. 6C) and horizontal (Fig. 6D) sections of the cerebellum. Blue light was illuminated onto lobule IXab for 5 seconds (50 Hz, 10 ms pulses, ∼50 mW/mm^2^), the same duration as in the previous study of electrical stimulation [16]. We first confirmed that SS firing was strongly activated during 5-sec light illumination (n = 10 units from 2 rats, Fig. 7A–C), and then tested the effects of photostimulation on BP. When lobule IXab was illuminated by blue light, a slight decrease in BP was observed (Fig. 7D). Normalized mean arterial pressure (nMAP) averaged across animals showed that BP was significantly lower during and after light illumination than that before light onset (*P*<0.01 at 5 sec, *P*<0.05 at 7.5 sec after light onset, one-way repeated-measures ANOVA followed by Bonferroni-corrected *t*-test, n = 6 rats, Fig. 7E). The magnitude of the depressor effect induced by photostimulation (∼1.3% fall) was comparable to that induced by electrical stimulation of lobule IXab using 200 µA current in anesthetized rats (Fig. S1). The effect of ChR2-mediated photostimulation of lobule IXab on BP was significantly larger than that of blue light illumination onto lobule IXab injected with PBS or ChR2-mediated photostimulation of neighboring lobule VIII (*P*<0.05 by Tukey-Kramer multiple comparison test, n = 3 for PBS and n = 4 for lobule VIII, Fig. 7F). These results demonstrate that activation of PCs within lobule IXab leads to depressor responses in anesthetized rats.

### eNpHR-mediated photoinhibition of PCs in the uvula provokes pressor responses

**Figure 8:**
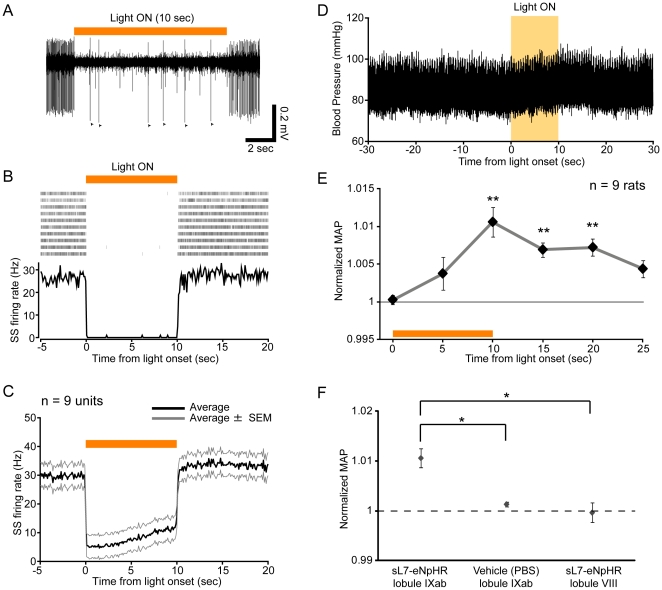
The effects of eNpHR-mediated inhibition of PCs in lobule IXab. (A) Ten sec continuous photoinhibition (indicated by a horizontal orange bar) of lobule IXab decreased SS firing rate of a PC. All of spikes observed during light illumination were CSs in this trial. Arrowheads indicate CSs. (B) Responses of SSs to photoinhibition. Shown at the top is a spike raster plot (10 trials are shown in horizontal row), and at the bottom is a PSTH, averaged across all trials (bin width, 100 ms). The intertrial interval was 90 sec. Period of photoinhibition is indicated by an orange bar. (C) Averaged response of SSs. Black line indicates average, and gray lines indicate average ± SEM. n = 9 units, 2 rats. (D) A sample raw trace of arterial blood pressure around the time of photoinhibition. (E) BP response to photoinhibition averaged across rats. **P<0.01, one-way repeated measures ANOVA followed by Bonferroni-corrected *t*-test. Width of time window used for calculating nMAP values at each time point is 1 sec. n = 9 rats. (F) The effects of photoinhibition of lobule IXab on BP were compared with that of orange light illumination onto vehicle (PBS)-injected lobule IXab (n = 3 rats) and photoinhibition of lobule VIII (n = 3 rats). nMAP values averaged during the period from 9 to 10 sec after light illumination onset were plotted. *P<0.05, Tukey-Kramer multiple comparison test.

We next examined the effects of eNpHR-mediated photoinhibition of PCs on resting BP in sL7-eNpHR rats. Orange light was continuously illuminated onto lobule IXab for 10 seconds (∼50 mW/mm^2^). During this period, SS firing was almost completely inhibited in the representative PC shown in Fig. 8A and 8B. CSs still fired during light illumination (Fig. 8A). The population average of SS responses to orange light showed that decrease in SS firing rate was prominent immediately after light onset, then gradually increased until light offset, and then returned to the baseline level after light offset (n = 9 units from 2 rats, Fig. 8C).

We then tested the effects of eNpHR-mediated photoinhibition on BP. When lobule IXab was illuminated by orange light, a slight increase in BP was observed (Fig. 8D). nMAP data averaged across animals showed that BP was significantly higher during and after light illumination than that before light onset (*P*<0.01 at 10, 15 and 20 sec from light onset, one-way repeated-measures ANOVA followed by Bonferroni-corrected *t*-test, n = 9 rats, Fig. 8E). The effect of eNpHR-mediated photoinhibition of lobule IXab was significantly larger than that of orange light illumination onto lobule IXab injected with PBS or eNpHR-mediated photoinhibition of neighboring lobule VIII (*P*<0.05 by Tukey-Kramer multiple comparison test, n = 3 for PBS and lobule VIII, Fig. 8F). These results demonstrate that inhibition of PCs within lobule IXab of anesthetized rats leads to pressor responses, opposite those to ChR2-mediated photostimulation.

## Discussion

In this study, we developed a method for manipulating PC activity using a lentivirus-based optogenetic technique, and then applied it to the analysis of cerebellar cardiovascular control. ChR2 or eNpHR was efficiently expressed in PCs under the control of the shortened L7 promoter. All of the light-responsive PCs (23 out of 23 cells) in sL7-ChR2 rats were activated by blue light, and almost all of the light-responsive PCs (30 out of 32 cells) in sL7-eNpHR rats were inhibited by orange light. Using this technique, we showed that the activation and inhibition of the cerebellar uvula exerted opposite effects on resting BP. This new method of cerebellar cortex stimulation and inhibition is useful for probing the functions of specific cerebellar areas, since it can be used to manipulate the output of the cerebellar cortex alone, without affecting afferent fibers reaching the cerebellar cortex.

The L7 gene promoter has been extensively utilized for cerebellar PC-specific transgene expression, mainly in transgenic mice [14], [19]. The sL7 promoter used in this study had a 0.3 kb sequence upstream of the transcription start site, and included only a part of the structural gene extending from the transcription start site to upstream of the first ATG within exon 2. We used this short promoter because lentiviral titer depends on the size of packaged inserts, heavily decreasing for large packaged inserts [25]. In a previous study, Mandolesi et al. used an ‘L7 minigene’, consisting of a 1 kb L7 promoter, 2 exons, and 1 intron, for transgene expression using a lentiviral vector [26]. They showed that GFP expression induced with the L7 minigene was observed mainly in PCs, with ectopic expression also observed in Golgi cells. The L7 promoter sequence of the sL7 promoter is shorter than that used by Mandolesi et al., although the expression pattern of ChR2-EYFP induced by the sL7 promoter was consistent with their observation, since ChR2-EYFP expression in a small number of Golgi cells was also observed in sL7-ChR2 rats (Fig. 2). Although the reason why the PC specificity of eNpHR-EYFP was lower than that of ChR2-EYFP is unclear, it is possible that the sequence of the eNpHR-EYFP gene interacts with L7 promoter activity in a way that promotes ectopic eNpHR-EYFP expression outside PCs [27]. Another possibility is that eNpHR-EYFP expression was affected by the transcription of upstream genes within the non-target cell genome into which the lentiviral genome was inserted [28].

Although ChR2-mediated photostimulation and eNpHR-mediated photoinhibition strongly influenced SS activity, they influenced on the CS activity very little (Fig. 2E, 3B). The mechanism of CS generation is not yet fully understood [29]; however, the following may explain our results. First, because synaptic currents evoked by climbing fiber are quite large [24], it is possible that photocurrents induced by ChR2 and eNpHR were not sufficient to evoke or inhibit CSs. In addition to the magnitude of the current, the waveform of the stimulation current may also be important for the induction of CSs [30]. Because somatic square current pulse injection into a PC evokes spike bursts instead of physiological CSs [31], and our photostimulation protocol would induce photocurrents like square current pulses, rather than climbing fiber-evoked synaptic conductance, it is plausible that ChR2-mediated photostimulation cannot evoke physiological CSs. However, even if ChR2-mediated photostimulation and eNpHR-mediated photoinhibition cannot directly evoke or inhibit CSs, it is also possible that an enhanced or an inhibited SS firing indirectly affects CS firing through the feedback of the cerebellar cortico-nuclear-olivary circuit. Indeed, Miall et al. found that a subtle enhancement of SS firing ∼150 ms before CS firing during spontaneous firing of PCs [32]. It is possible that we were unable to detect a subtle effect on CS firing under our experimental conditions, because CSs occur at low frequencies of approximately 1 Hz *in vivo* [33].

In a previous study of mouse cerebral cortex expressing ChR2 under the control of EF1α, it was shown that some portions of ChR2-negative pyramidal neurons were indirectly activated by light [34]. Lima et al. suggested that one potential source of indirect activation is polysynaptic activation through recurrent excitatory connections including ChR2-positive neurons. In cerebellar cortex, PCs are presumed to receive inhibitory input from recurrent axon collaterals of nearby PCs activated by ChR2 [35]. In the present study, all of the light-responsive PCs in sL7-ChR2 rats increased SS firing rate in response to blue light (Fig. 4F). This finding suggests that the indirect inhibition by recurrent axon collaterals was not sufficient to inhibit the activity of ChR2-expressing PCs in the presence of direct activation of it by ChR2. In the case of eNpHR-mediated photoinhibition, we found that a small percentage of PCs (2 out of 32 cells) increased SS firing rate in response to orange light (Fig. 5C). There are several possible reasons for the PC excitation by eNpHR-mediated photoinhibition. First, inhibition of recurrent axon collaterals of PCs would lead to disinhibition of PC activity. Second, inhibition of stellate/basket cells would also lead to disinhibition of PC activity. Third, inhibition of Golgi cells would lead to disinhibition of discharge from granule cells and eventually facilitate PC firing [36]. Ectopic eNpHR expression in stellate/basket and Golgi cells observed in sL7-eNpHR rats (Fig. 3F, L, M, N) suggests the possibility that PC excitation by eNpHR-mediated photoinhibition was due to stellate/basket inhibition and/or Golgi inhibition. Nevertheless, since most light-responsive PCs in sL7-eNpHR rats decreased SS firing rate in response to orange light (Fig. 5C), overall PC activity within lobule IXab of sL7-eNpHR rats appears to have been strongly inhibited by orange light.

ChR2-mediated photostimulation of the uvula caused ∼1.3% decrease in BP, which was comparable to the effect of electrical stimulation at a current strength of 200 µA (Fig. S1). The effective current spread from an electrode tip can be expressed by *r = (I/K)^1/2^*, where *r* is the distance of effective current spread from the electrode tip in mm, *I* is the current in µA, and *K* is the current-distance constant at an average of 1292 µA/mm^2^ for cortical pyramidal cells when using 200 µs duration pulses [37]. If this formula is applied to our electrical stimulation experiments in the cerebellar cortex, PCs directly activated by 200 µA electrical stimulation are located within a sphere with a radius of ∼0.39 mm centered at the electrode tip (surface area is ∼1.9 mm^2^). Because the density of PCs in the rat cerebellum is 1.0×10^3^ per mm^2^ [38], the number of PCs activated by 200 µA electrical stimulation was less than 1.9×10^3^. In ChR2-mediated photostimulation experiments, a blue laser was illuminated onto the cerebellar surface of lobule IXab (∼50 mW/mm^2^ at the cerebellar cortical surface), and the diameter of the beam spot on the cortical surface was ∼1.0 mm. Most PCs located deeper than 1.0 mm from the cerebellar surface were ChR2-EYFP-negative in our injection method (Fig. 6). Assuming that the light intensity is enough to activate ChR2-expressing neurons (>1 mW/mm^2^, [39]) at 1.0 mm from the cortical surface, the number of PCs activated by ChR2-mediated photostimulation is less than 4.7×10^3^ [(the density of PCs)×(the surface area of a cylinder with a radius of 0.5 mm and with a height of 1.0 mm)]. This number is, at least on its order, comparable with that of electrical stimulation, suggesting that the efficiency of PC stimulation is not so different between electrical and photic stimulations.

The influence of the cerebellar uvula on cardiovascular control has been studied in rabbits and cats [5], [16], [17], [40], [41]. PCs in this region project to the lateral parabrachial nucleus, through which they connect to the solitary tract nucleus (NTS) [17]. Because NTS receives primary afferent fibers from baroreceptors and plays a fundamental role in baroreceptor reflexes, it has been suggested that cardiovascular responses caused by uvular stimulation are elicited through these connections [5]. In this study, we showed that the electrical stimulation of lobule IXb decreased BP in urethane-anesthetized rats as well as in cats and rabbits (Fig. S1), suggesting that the cardiovascular regulatory function of the uvula is preserved in rats. However, the magnitudes of depressor responses were smaller in rats than in cats and rabbits, despite similar stimulation parameters [16], [40]. In the previous study performed using rabbits, it was shown that the electrical stimulation of lobule IXb evokes depressor responses by more than 20% under urethane anesthesia [16]. Since our electrical stimulation experiments in rats were also performed under urethane anesthesia, the discrepancy between magnitudes of effects obtained in our study and those obtained in the previous studies is not likely to be due to the difference in anesthesia. Moreover, the difference in the stimulus (electrical stimulation vs. optogenetics) will not explain this discrepancy, as well, because the number of PCs activated by electrical stimulation and that activated by ChR2-mediated photostimulation to evoke depressor responses of the same magnitudes was not so different, as described above. Therefore, the cause of the discrepancy would be mainly due to the species difference (rats vs. cats and rabbits). PCs within the cerebellar uvula also project to the inferior and medial vestibular nuclei, which mediate vestibular effects on the sympathetic nervous system during postural alteration [42]. The uvula is therefore believed to regulate vestibular effects on cardiovascular responses during changes in posture [42]. A possible explanation for the species difference in uvular influence on BP is the length of the body, because the stress to the cardiovascular system during postural alteration is dependent on the length of the body, and would be more severe in animals with large bodies [43]. It is plausible that the cerebellar uvula affects more robustly the cardiovascular system in cats and rabbits than in rats.

Although electrical or chemical stimulation of the medial area of the uvula evokes a pattern of cardiovascular responses that consists of an increase in BP and heart rate accompanied by vasoconstriction in unanesthetized decerebrate cats and rabbits, stimulation of the same area in anesthetized rabbits yields the opposite responses, i. e., a decrease in BP and heart rate accompanied by vasodilation [5], [16]. Because of this discrepancy, it is still unclear how PC activity in the uvula affects the cardiovascular system. In the present study, we showed that ChR2-mediated photostimulation of lobule IXab decreased BP, while eNpHR-mediated photoinhibition increased it (Fig. 7E, 8E). We also showed that photostimulation and photoinhibition of lobule VIII, the neighboring lobule of IX, had no effect on BP, which is consistent with the result of electrical stimulation of lobule VIII in rabbits [16]. Blue or orange light illumination onto PBS-injected lobule IXab itself didn't affect BP (Fig. 7F, 8F). Electrophysiological recordings showed that ChR2-mediated photostimulation increased population PC activity (Fig. 7C) and eNpHR-mediated photoinhibition decreased it (Fig. 8C). Therefore we can conclude that increase or decrease in PC activity in the uvula of anesthetized rats leads to depressor or pressor responses, respectively. And these results demonstrate that causal relationships between local PC activity and functions of the cerebellum can be elucidated by using the lentivirus-based optogenetic method we developed here.

## Materials and Methods

### Ethics statement

All procedures were performed in accordance with a protocol approved by the University of Tokyo Animal Care Committee (the permit number is MED: P09-092). All surgical procedures were performed under anesthesia, and all efforts were made to minimize suffering and number of animals.

### Plasmid construction

The sL7 promoter, comprising a 1 kb sequence upstream of the first ATG within exon 2 of L7 gene, was cloned by polymerase chain reaction (PCR) from the template plasmid, pL7-tWGA (kindly provided by Dr. Y. Yoshihara, RIKEN Brain Science Institute, Wako, Japan). A half nanogram of plasmid template was amplified in a 25 µl reaction mixture containing 0.2 µM of each primer, 200 µM of each dNTP, 1×PrimeSTAR buffer and 0.625 U PrimeSTAR HS DNA polymerase (Takara Shuzo, Co., Ltd., Shiga, Japan). The primer combinations were as follows: sense 5′-GTT CCA CCC TCA TGT TGG TTG-3′ and antisense 5′-CGA TCG CCC TGC ACG TG-3′. PCR was performed at 98°C for 5 min and then for 35 cycles at 98°C for 10 sec, 60°C for 5 sec and 72°C for 1 min in a Program Temp Control System PC-800 (ASTEC, Fukuoka, Japan). The PCR product was electrophoresed on agarose gel (1% w/v), extracted using QIAquick Gel Extraction Kit (QIAGEN, Hilden, Germany), subcloned into pGEM-T Easy Vector (Promega, Madison, WI, USA) and sequenced.

For generation of lentiviral transfer vectors, pCL20c sL7-hChR2-EYFP-WPRE, and pCL20c sL7-eNpHR-EYFP-WPRE, SpeI/XbaI-digested hChR2-EYFP fragment from pcDNA3.1-hChR2-EYFP or BamHI/EcoRI-digested eNpHR-EYFP fragment from pLenti-CaMKIIα-eNpHR-EYFP-WPRE (both plasmids were kindly provided by Dr. K. Deisseroth, Stanford University) were inserted into EcoRI/NotI-digested pCL20c MSCV-GFP (kindly provided by Dr. Arthur W. Nienhuis, St. Jude Children's Research Hospital, TN, USA) [44] by blunt-end ligation to replace the EGFP coding sequence. The ClaI-digested WPRE fragment from CS-CA-MCS (kindly provided by Dr. H. Miyoshi, RIKEN BioResource Center, Ibaraki, Japan) [45] was subcloned into the ClaI site of pCL20c MSCV-hChR2-EYFP or pCL20c MSCV-eNpHR-EYFP. The EcoRI-digested sL7 promoter fragment was inserted into MluI/EcoRI-digested pCL20c MSCV-hChR2-EYFP-WPRE by blunt-end ligation to replace the MSCV promoter. The NcoI/SalI-digested sL7 promoter fragment was also inserted into MluI/AgeI-digested pCL20c MSCV-eNpHR-EYFP-WPRE by blunt-end ligation to replace the MSCV promoter.

### Lentiviral preparation and injection

Human embryonic kidney (HEK) 293T cells (obtained from RIKEN BioResource Center, Cell No. RCB2202) were cultured in Dulbecco's modified Eagle's medium (Invitrogen, CA, USA) supplemented with 10% fetal bovine serum at 37°C in a 5% CO_2_ atmosphere, and lentiviral vectors were prepared as described previously [46]. We produced two lentiviral vectors, Lenti-sL7-hChR2-EYFP-WPRE (sL7-ChR2) and Lenti-sL7-eNpHR-EYFP-WPRE (sL7-eNpHR), in this study (Fig. 1). Titers of virus stocks were determined by the DNA titration method [47]. Serial dilutions of each virus preparation were added to 4×10^5^ HEK293T cells growing in a monolayer in six-well plates in a total volume of 2 ml of culture medium. After 24 hours, the medium was changed and culture was continued for another 2 days. Then the genomic DNA (gDNA) from approximately 1–10×10^5^ vector transduced cells was isolated using FastPure DNA Kit (Takara Shuzo, Co., Ltd.). TaqMan® MGB Probe pWPRE (5′-FAM-CAT GGC TGC TCG CCT-MGB-3′), and the primers WPRE-F (5′-CGG CTG TTG GGC ACT GA-3′) and WPRE-R (5′-GAG GGC CGA AGG GAC GTA-3′) for quantitative real-time PCR analysis were designed using the Primer Express software program and obtained from Applied Biosystems (CA, USA). PCR reaction and data analysis were performed using *StepOne*™ Real-Time PCR system (Applied Biosystems). The gDNA was diluted and 2 µl of DNA solution was mixed with 18 µl of a PCR master mix consisting of 1×TaqMan® Fast Universal PCR Master Mix, 0.9 µM of each forward primer (WPRE-F) and reverse primer (WPRE-R), and 0.25 µM of pWPRE. For negative controls, 2 µl of gDNA solution from non-transduced cells were used. Amplifications were performed using one cycle of 95°C for 20 sec, 40 cycles of 95°C for 1 sec and 60°C for 20 sec. Amplification of WPRE sequence-containing vector plasmid DNA for generation of a standard curve was performed using concentrations of plasmids ranging from 10^7^ molecules/µl to 10^2^ molecules/µl as determined by spectrophotometry (Ultrospec, model 4000, GE Healthcare, Buckinghamshire, UK). Plasmid DNA used for standard curve generation was diluted with the gDNA from uninfected cells to control for any inhibitory effect of gDNA on PCR. All reactions were carried out in duplicate. The number of vector DNA molecules in transduced cells was calculated by comparing threshold cycle values (C_T_) of samples to that of the plasmid standard curve. Copy numbers of gDNA was estimated using TaqMan® Copy Number Reference Assays (Applied Biosystems). In determining the final DNA titers of vectors, the total number of vector DNA molecules in transduced cells was corrected for dilution and copy number of gDNA.

Eight- to twelve-week-old male Wistar rats (Nihon SLC, Shizuoka, Japan) were used for lentiviral or PBS injection. Each rat was anesthetized with ketamine/xylazine (90 mg/kg and 10 mg/kg, respectively), and positioned in a stereotaxic apparatus (SR-6R; Narishige, Tokyo, Japan). A midline sagittal incision was made, and the skull over the cerebellum was exposed. For targeting of vector solution to lobule IXab or VIII, a small hole was drilled 14.0 mm posterior to bregma and on the midline. A glass pipette (∼50 µm tip diameter) was attached to a 32-gauge blunt end needle, and the needle was then attached to a 10 µl gas-tight Hamilton syringe (Hamilton Company, NV, USA). The glass pipette was tilted 20° forward in the sagittal plane, and lowered into the cerebellum to a depth of 4.1 mm (for IXab targeting) or 2.85 mm (for VIII targeting) from the dura mater [48]. Then virus solution (6 µl), at a titer of >10^10^ genome copies/ml, was injected at a flow rate of 200 nl/min using a micropump (UltramicroPump III; World Precision Instruments, FL, USA) and microprocessor-based controller (Micro4; WPI). The needle was left in place for an additional 10 min before being retracted from the brain. The scalp incision was sutured, and the rat was returned to a standard cage after recovering from the anesthesia. Lentiviral injection into lobule VI was performed as previously described [46]. Rats injected with a lentiviral vector into lobule IXab (sL7-ChR2 rats, n = 8; sL7-eNpHR rats, n = 11) were used for immunohistochemical, electrophysiological and blood pressure experiments. Rats injected with a lentiviral vector into lobule VI (sL7-ChR2 rats, n = 2; sL7-eNpHR rats, n = 5) or lobule VIII (sL7-ChR2 rats, n = 4; sL7-eNpHR rats, n = 3) were used only for electrophysiological or blood pressure experiments, respectively. Rats injected with PBS into lobule IXab (n = 3) were also used for blood pressure experiments. Animals were maintained for more than 8 days before use in physiological experiments. All procedures were performed in accordance with a protocol approved by the University of Tokyo Animal Care Committee.

### Immunohistochemistry and cell counting

Rats were perfused with saline followed by 4% paraformaldehyde in phosphate buffer. The brains were post-fixed in 4% paraformaldehyde for 2–4 h and sunk in 20% sucrose in PBS. A fluorescent stereoscopic image of whole brain was obtained using a cooled CCD-camera (VB-7000, Keyence, Osaka, Japan) attached to a fluorescence stereoscopic microscope (VB-G05, Keyence). For fluorescent staining, 25 µm thick sections were cut with a cryostat. Sections were blocked with 5% normal goat serum (WAKO, Osaka, Japan) and incubated for 24 h at 4°C with one of the following antibodies: mouse anti-parvalbumin monoclonal antibody (1∶4000, Sigma-Aldrich, MO, USA), mouse anti-calbindin-D28k monoclonal antibody (1∶3000, Sigma-Aldrich), mouse anti-NeuN monoclonal antibody (1∶1000, Millipore, MA, USA), or mouse anti-mGluR2 monoclonal antibody (1∶500, kind gift from Dr. R. Shigemoto, National Institute for Physiological Sciences, Japan) [21]. Then the sections were incubated for 3 h at room temperature with AlexaFluore647-conjugated goat anti-rabbit antibody (1∶500, Invitrogen). For staining of the representative sagittal and horizontal sections shown in Fig. 6, rabbit anti-GFP polyclonal antibody (1∶5000, Medical & Biological Laboratories, Aichi, Japan) was used to enhance EYFP signals. These sections were then incubated for 3 h at room temperature with AlexaFluore488-conjugated goat anti-rat IgG antibody (1∶500, Invitrogen), and counterstained with Hoechst 33258 (1 mg/ml, MolecularProbes, Eugene, OR, USA). The stained images were obtained using a fluorescence microscope (BZ-9000, Keyence).

For counting of particular cell type, three sagittal sections (separated from each other by 100 µm) around the center of the injection site were used for each rat. To calculate the percentage of each cell type among all EYFP-positive cells, we used sections stained with anti-parvalbumin antibody. We counted all EYFP-positive cells in each section, and then counted EYFP-positive stellate/basket cells and PCs. Stellate/basket cells and PCs were discriminated by their morphology and the locations of cell bodies in the cerebellar cortex. EYFP-positive cells considered to be neither stellate/basket cells nor PCs were designated ‘other’. Then the numbers counted were summed across three sections and percentages were calculated by dividing the number of EYFP-positive cells of each type by that of all EYFP-positive cells. To measure the transduction efficiencies into each cell type, EYFP-positive cells were counted and scored for co-labeling with each cell type marker, calbindin-D28k (PC), parvalbumin (stellate/basket cell), NeuN (granule cell) or mGluR2 (Golgi cell). Cell counting for calculation of transduction efficiencies was performed within the surface area (0–500 µm depth from cortical surface) of lobule IXab in each section.

### Electrophysiological recording and optical stimulation

Each rat injected with a lentiviral vector into cerebellar lobule VI, or IXab was anesthetized with ethyl carbamate (1.2 g/kg) and then positioned in a stereotaxic apparatus. The skull around the virus-infected area of each rat was exposed and carefully removed by a drill. The activity of single neurons was recorded extracellularly from the infected area using a glass-coated tungsten microelectrode (resistance<0.6 MΩ). The microelectrode was advanced through the intact dura mater into the cortex using a hydraulic micromanipulator (MO-10, Narishige). Neural signals were amplified using an amplifier (AB651J, NIHON KOHDEN, Tokyo, Japan) and band-pass filtered (0.05–5 kHz), and then digitized at 25 kHz and stored using the Recorder Software (Neural Data Acquisition System, Plexon, TX, USA). Stored data were analyzed offline with the ‘Off-line Sorter’ program (Plexon) for sorting into single-unit data by waveform analysis. Single-unit data were then analyzed with MATLAB (Mathworks, MA, USA). A neural signal was considered to originate from a PC if it exhibited two types of spiking activity: simple spikes (SSs) characterized by a single depolarization occurring between 20 and 200 Hz and complex spikes (CSs) characterized by an initial fast depolarization followed by smaller and relatively constant spikelets [49]. Simple and complex spikes were judged to have originated from the same PC when a transient pause (∼20 ms) in SS firing followed each CS.

The blue light source was a diode pumped solid state (DPSS) laser (peak wavelength at 473 nm, 200 mW; beam diameter, 2.0 mm; CNI Optoelectronics, Changchun, China). The outgoing beam was passed through an ND filter (Thorlabs, NJ, USA) to calibrate the output power and then an electrically controlled mechanical shutter (hole diameter, 2.0 mm; Model LS3, UNIBLITZ, NY, USA) to control the length of each light pulse. The mechanical shutter was controlled by an electronic stimulator (NIHON KOHDEN). The beam was next entered into an optic fiber (core diameter, 62.5 µm) through a fiber collimator. The laser, ND filter, shutter and collimator were mounted on an optics breadboard. The output beam from the optic fiber was also passed through another fiber collimator (focal length, 4.50 mm; beam waist diameter of output beam, 0.9 mm). The power of the output beam was measured with a power meter (PM100D, Thorlabs). The optic fiber was held in a micromanipulator to control the position of the beam spot on the surface of the cerebellum. The orange light source was another DPSS laser (peak wavelength at 593 nm, 200 mW; beam diameter, 3.0 mm; CNI Optoelectronics). The outgoing beam was passed through an ND filter. Then the beam was passed through two lenses (focal length: 1^st^ lens 60 mm, 2^nd^ lens 30 mm) to thin the beam diameter from 3.0 to 2.0 mm. The beam was next entered into an optic fiber through a fiber collimator. The output beam from fiber collimator was also passed through another collimator (focal length, 4.50 mm; beam waist diameter, 0.9 mm). The tip of fiber and cerebellar surface was separated at approximately 8 cm. The divergent angle of the output beam was 0.045°. Therefore the beam spot diameter on the cerebellar surface was calculated as approximately 1.0 mm. Light intensity was set at ∼50 mW/mm^2^ in all electrophysiological experiments [50].

The effect of optical stimulation on each PC's firing rate was heard on a sound monitor. It was therefore possible to roughly determine the effective site of stimulation for each cell by ear. To characterize the responses of PCs to blue or orange light in sL7-ChR2 or sL7-eNpHR rat cerebellum, we used a light pulse of 100 ms duration with the interstimulus interval set at 500 ms. We performed 100 trials for each cell. Light-responsive PCs were classified by performing a paired *t*-test between the SS firing rate during the 50 ms period before light onset and that during the 50 ms period after latency across all trials for that cell, and thresholded at the *P*<0.05 significance level [51]. To determine the latency between light onset and neural response, a 6 ms-long sliding window was swept through the electrophysiology data to search for the earliest 6 ms period that deviated from baseline SS firing rate (which was calculated from the SS firing rate during the 6 ms period before light onset), as assessed by paired *t*-test between the SS firing rate during each window and that during the baseline period, across all trials for each cell. An SS firing rate modulation index (MI) was calculated using following equation: (firing rate during the 50 ms period before light onset)/(firing rate during the 50 ms period after latency).

### Arterial blood pressure measurement

Each rat injected with a lentiviral vector into lobule IXab or VIII was anesthetized with ethyl carbamate (1.2 g/kg). Body temperature was maintained at 37.5°C using a homeothermic heating pad (BioResearch Center, Aichi, Japan). A catheter (inner diameter, 0.5 mm; outer diameter, 0.9 mm; polyethylene tube SP35, NATSUME SEISAKUSHO, Tokyo, Japan) was inserted into the left femoral artery and connected to a blood pressure transducer (DX-360, NIHON KOHDEN) to measure BP continuously [13]. BP signals were amplified using a blood pressure amplifier (AP-641G, NIHON KOHDEN), digitized at 1 kHz and analyzed offline. Mean arterial pressure (MAP) was calculated as diastolic pressure plus one third of the pulse pressure for each beat. To calculate normalized MAP (nMAP) in each trial, average MAP during 0–5 sec before light onset was used.

For light illumination onto lobule IXab or VIII, the skull around the lobule IX or VIII of each rat was exposed and carefully removed. Then a tungsten microelectrode was positioned at lobule IXb (AP, 15.4 mm; ML, 0.0 mm; DV, 6.0 mm from bregma) or lobule VIII (AP, 15.4 mm; ML, 0.0 mm; DV, 3.6 mm from bregma) and used to record PC activity. The position of light illumination was then adjusted so as to maximize the effect of light illumination on that cell.

To investigate the effects of ChR2-mediated photostimulation on resting BP, blue light was illuminated for 5 sec at 50 Hz (10 ms light/10 ms interval) in each trial. Blue light intensity was ∼50 mW/mm^2^. The intertrial interval was at least 1 min. We performed at least 20 trials per each rat.

To investigate the effects of eNpHR-mediated photoinhibition on BP at rest or during postural alteration, orange light was illuminated for 10 sec continuously in each trial. Orange light intensity was ∼50 mW/mm^2^. The intertrial interval was at least 1 min. We performed at least 30 trials per each rat.

For electrical stimulation experiments, we used 4 male Wister rats (10 weeks old). Each rat was anesthetized, and received surgery in the same way with lentivirus-injected rats. Electrical stimulation of lobule IXb was via a Platinum/Iridium microelectrode (0.2–0.3 MΩ, MicroProbes, MD, USA) using a monopolar 5 sec pulse train (0.2 ms, 50 Hz) of cathodal current. Stimulation sites were 0.5 mm depth from cortical surface at lobule IXb (AP, 15.4 mm; ML, 0.0 mm; DV, 6.0 mm from bregma). The intertrial interval was at least 1 min. We performed at least 30 trials for each current condition (100, 200, 300, 400 and 500 µA) in every rat.

### Statistics

All statistical analyses were performed using SAS/STAT (SAS institute, NC, USA) and MATLAB software. Tukey-Kramer multiple comparison test was used for histological and BP data analysis. One-way repeated measures ANOVA with the Bonferroni-corrected *t*-test was used to evaluate time-dependent effects of ChR2-mediated photostimulation and eNpHR-mediated photoinhibition of lobule IXab on BP. An error bar denotes SEM except when indicated otherwise.

## Supporting Information

Figure S1 **The effects of electrical stimulation of lobule IXab on BP.** Stimulus intensity-BP change relationship was examined for stimulus condition of 100, 200, 300, 400 and 500 µA (0.2 ms pulse, 50 Hz, 5 sec). nMAP values averaged during the period from 4 to 5 sec after stimulation onset were plotted for each current condition. *P*<0.001, one-way repeated measures ANOVA. *P<0.05, Bonferroni-corrected *t*-test (the null hypothesis stated that the mean was equal to 1). n = 4 rats.
(TIF)
