# Supplementary figures and images for: Optogenetic Manipulation of Cerebellar Purkinje Cell Activity In Vivo
Source: PLoS One. 2011 Aug 5;6(8):e22400. doi: 10.1371/journal.pone.0022400 (PMC3151259; doi:10.1371/journal.pone.0022400)

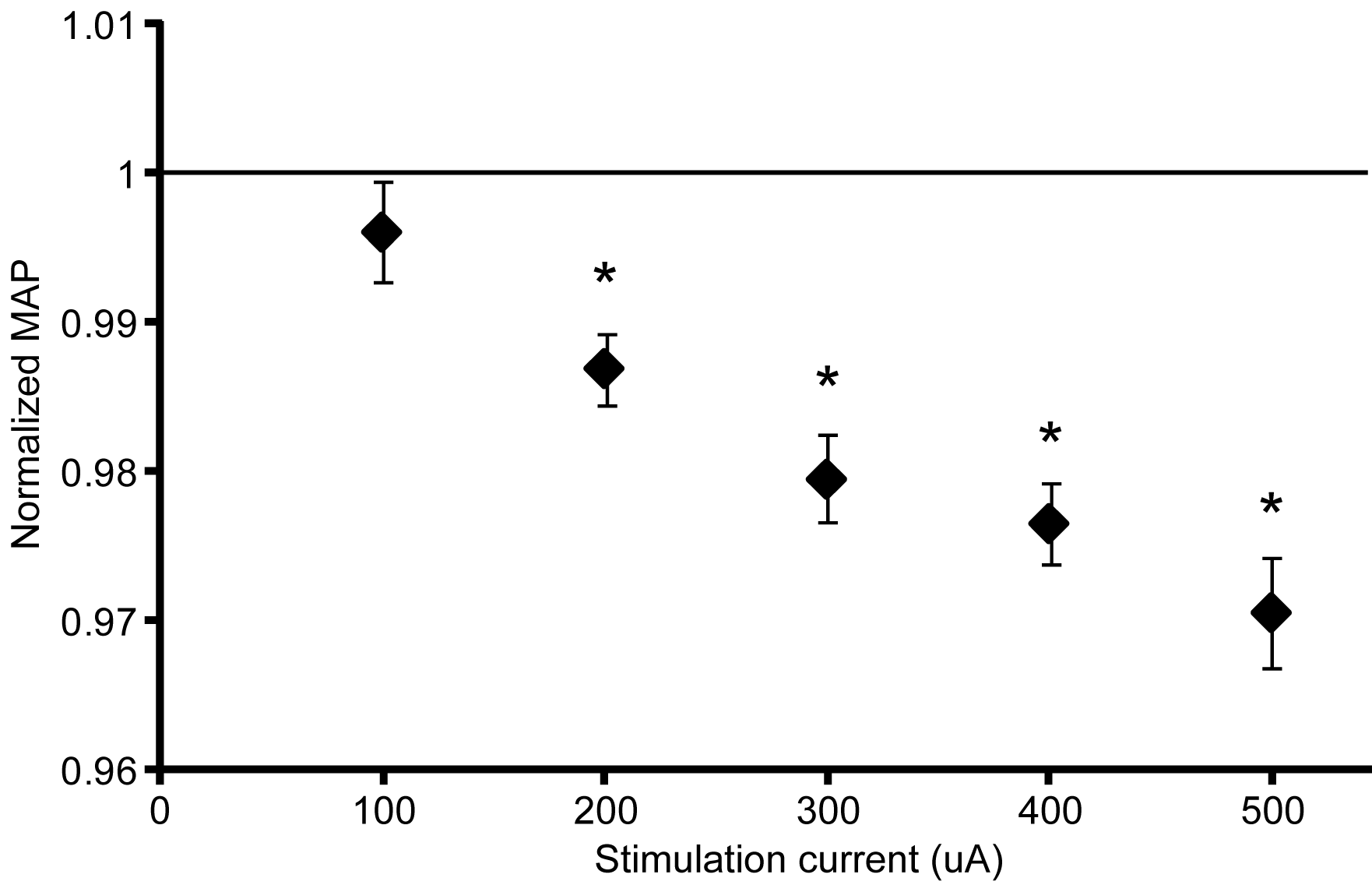

Supplement: Figure S1 — The effects of electrical stimulation of lobule IXab on BP. Stimulus intensity-BP change relationship was examined for stimulus condition of 100, 200, 300, 400 and 500 µA (0.2 ms pulse, 50 Hz, 5 sec). nMAP values averaged during the period from 4 to 5 sec after stimulation onset were plotted for each current condition. P<0.001, one-way repeated measures ANOVA. *P<0.05, Bonferroni-corrected t-test (the null hypothesis stated that the mean was equal to 1). n = 4 rats. (TIF) [file pone.0022400.s001.tif]
